# Relation between Body Humors and Hypercholesterolemia: An Iranian Traditional Medicine Perspective Based on the Teaching of Avicenna

**Published:** 2012-03-01

**Authors:** M Emtiazy, M Keshavarz, M Khodadoost, M Kamalinejad, S A Gooshahgir, H Shahrad Bajestani, F Hashem Dabbaghian, M Alizad

**Affiliations:** 1 School of Iranian Traditional Medicine, Tehran University of Medical Sciences, Tehran, Iran; 2 Research Institute for Islamic and Complementary Medicine, Tehran University of Medical Sciences, Tehran, Iran; 3 Department of Traditional Medicine, School of Medicine, Shahed University, Tehran, Iran; 4 Department of Pharmacognosy, School of Pharmacy, Shahid Beheshti University of Medical Sciences, Tehran, Iran; 5 Department of Endocrine and Metabolism, Rasoole Akram Hospital, Tehran University of Medical Sciences, Tehran, Iran

**Keywords:** Hypercholesterolemia, Iranian Traditional medicine, Stomach, Liver, Digestion

## Abstract

**Background:**

Cardiovascular diseases are among the most important causes of morbidity and mortality in the world. One of the important risk factors of cardiovascular disease is hyperlipidemia especially high levels of serum cholesterol. Due to the importance of hypercholesterolemia, being a serious condition, various treatments are used to control it, regardless of the cause, most of treatments, focused on reducing the level of serum lipids. This study aims to determine various view points for hypercholesterolemia in Iranian traditional medicine.

**Methods:**

We used several Iranian traditional medicine resources and literatures; then based on these texts; a pilot study was designed to assess their effects in 10 patients with high plasma cholesterol. The sign and symptoms in main digestive organs (Stomach and liver) were also evaluated.

**Results:**

Some patients showed hepatic temperament but all patients had gastric temperament.

**Conclusion:**

With reference to Iranian traditional medical texts and literatures, the organs involved in the process of digestion, particularly the stomach and the liver play the most important role. Yet the proper function of stomach as the first step involved in the digestion chain should be emphasized.

## Introduction

Lipids are body components which play important roles in energy storage and in the structure of the cell membranes, hormones and secondary mediators. Plasma lipid concentration depends on the balance between production, consumption and deposition of lipids.[1] The hyperlipidemia theory is based on the biochemical changes in the blood, i.e. disturbed lipid metabolism and as a consequence of increased concentration of lipids in the blood circulation.[2] Rising plasma lipid levels can lead to disorders such as atherosclerosis.[3][4][5][6] Atherosclerosis is a progressive disorder causally related to multiple cardiovascular diseases like coronary artery disease (CAD).[7][8][9][10][11] CAD is a leading cause of mortality, morbidity, and disability in the world.[12][13] The high prevalence and morbidity associated with CAD in Iran is one of the most pressing health problems.[14] One of the important risk factors which have been identified in connection with ischemic heart disease is the high level of serum cholesterol.[15][16][17][18][19][20][21] According to the American Heart Association report, 102.2 million Americans age 20 and older have total blood cholesterol levels of 200 milligrams per deciliter or higher.[22]

The important point in atherosclerosis disorder is to modify the level of lipids; because improved lipid levels to prevent atherosclerosis is the foundation.[23] There are different ways to treat hypercholesterolemia, such as diet therapy and drug therapy. Several studies indicated diet therapy not to be very promising[24] and drug therapy may have different complications.[25] Now, day-by-day several different synthetic drugs of better efficacy are being introduced. But apart from being effective, most of them induce adverse side effects.[25] For instance; statins, among the most commonly prescribed of medications which are used for the treatment of hypercholesterolemia and for the prevention of coronary heart disease[26] are related with various musculoskeletal side effects.[27] Therefore, these potential agents could not be used for a prolonged period of time but hyperlipidemia requires long term treatment.[2] So it is important to choose lipid lowering methods and medications that do not adversely affect their efficacy profile, or reinforce their potential negative side effects. Therefore, search for safe and effective cholesterol lowering methods was the main motivating factor behind this study. Today the world is moving towards integrating traditional and complementary medicine[28] with the mainstream medicine to increase efficacy and to decrease side effects and costs.[29] One of the oldest and richest school of traditional medicine is the Iranian traditional medicine.[30] Thus in this article, we presented hypercholesterolemia from the viewpoint of Iranian traditional medicine as has been known unani medicine. To understand this perspective, it is necessary to explain some principles.

The Iranian traditional medicine system makes effort to propose the best possible ways by which a person can lead an optimum healthy life with least illness. The Iranian traditional medicine framework is based on some principals. One of the important principles is "Umore Tabiya". Umore Tabiya includes different parts: Arkan (Rokn), Amzaj (Mizaj, Temperament), Akhlat (Khelt, humour), Aza (Ozv), Arvah (Rooh), Qova (Qova) and Afal (Fel), respectively.[31][32][33]

"Mizaj" (temperament) is a quality which is a consequence of mutual interaction of the four contradictory primary qualities (Hot, Cold, Wet, Dry) residing within the elements. These elements are so meticulously intermixed with each other that they lie in a very intimate relationship to one another. Their opposite powers intermittently conquer and are conquered until a state of balance is reached which is uniform throughout the whole. This result was given the name of temperament (Mizaj).[34][35][36] In other words, Mizaj means the dominant quality of the composite object.

Mizaj is one of the most important canons of Iranian traditional medicine system. It has an important function in maintaining the ideal healthy state of an individual. Vulnerability of its altered temperament which is called dystemperament (Sui' e Mizaj) leads to several different types of diseases.[37]

"Humor" which is called in the Iranian traditional medicine texts as "Khelt" is a wet and fluid substance which foodstuffs in the first stage of permutation changes to it. Normally there are four humors in the human body: "Phlegm or Balgham, Blood or Dam, Yellow bile or Safra and Black bile or Sauda". Each of the humors was related with pairs of qualities including cold and wet, hot and wet, hot and dry, and cold and dry, respectively.[38][39][40][41] In addition, each of humor in the characteristics is similar to one of the four elements: water, air, fire and earth, respectively.[42][43][44]

### Lipids and Humors 

In the Iranian traditional medicine texts, there is no concept of hypercholesterolemia as such; but in many cases has been described it as a disorder. As far as the presence of fat (Lipids) is concerned in blood, Ibn Sina (Avicenna) an ancient Iranian traditional physician[45][46][47][48] has reported its existence in blood, produced from "Dosoomat Al-Dam".[2][49] "Dosoomat" means "fatty, oily"[50] and "Dam" means "blood".[51]

Dosoomat of blood or the oily substance could be the lipids but as the biochemical analysis of blood was not available and they could not describe it as per modern parameters.2 According to the fundamentals of Iranian traditional medicine, the blood circulating in the vessels is a combination of four humors.[52] Therefore, the clues of blood lipids should be sought between the humors. Thus to clarify this issue, it is necessary to describe the production path of the humors.

### The Digestion Pathway: Humor Production

Based on available evidences and the detailed descriptions of the Iranian traditional medicine resources, ingested food undergoes various stages of digestion before reaching the tissues.[53][54] Ibn-Sina (Avicenna) and most of the Hakims (The wise master physicians of Iranian traditional medicine), are all of the opinion that digestion is a continuous process taking place from the mouth to the tissue that can be divided into four sequential stages10 including the gastric digestion stage, the hepatic digestion stage, the vascular digestion stage and the tissue digestion stage.

**Fig. 1:**
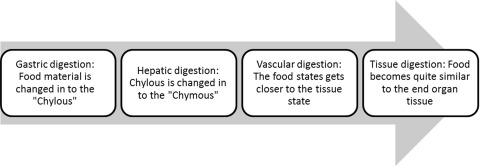
Four stages of digestion

Each stage of the digestion is composed of specific processing of the food material that must be carried on until it becomes suitable for used by the body. In each digestion process, the following actions take place (Figure 1):[55]

1. In the gastric digestion, some of the traits and characteristics of food material changes and appropriate absorbable material named "Chylous" is absorbed via the mesenteric vessels to the liver for further digestion.

2. In the hepatic digestion stage, the chylous is changed in to the "Chymous" that is composed of four humors (blood, phlegm, yellow and black bile), which will circulate in the vessels.

3. In the vascular digestion stage, the food state gets closer to the tissue state.

4. During the tissue digestion stage, food becomes quite similar to the end organ tissue.

As it was expressed, the humors are the final products of the hepatic digestion and in order for the humors, to be of good quality two conditions must be met including (i) Normal liver function for proper digestion and (ii) The (Gastric chylous), which is used by the liver for the production of humors that must have an appropriate composition. The second condition would not be present unless the stomach function properly and the ingested food is of good quality.

### Abnormal Humour Production Results in Lipid Disorders

According the book "The Qanon of Medicine" (by Avicenna), the foundation of Iranian traditional medicine system was based on the balancing humors in the human body.[56][57] Their imbalance caused several different diseases whereas restoration of the balance led to health.[56][57] Based on the Al-Qanun fi al-Tibb, the blood and what flows within the vessels (such as plasma lipids) are the product of the second digestion.[52] Thus, high plasma lipid conditions may be due to dysfunction of one or two of the previous digestion stages (Hepatic and gastric digestion stages).

**Fig. 2:**
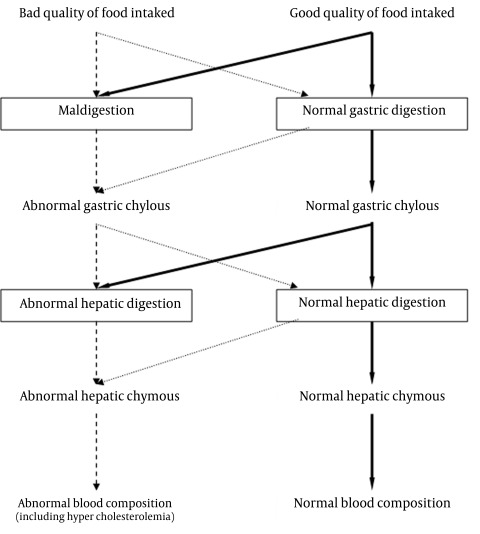
Different pathways of causing abnormal blood composition (including hypercholesterolemia).

As it was expressed previously, the continuity between the stages of digestion and even the organs involved is such that any disorder in one of them would bring about a disorder in other organs and stages. Abnormal vascular content may be a consequence of hepatic maldigestion. Also the hepatic maldigestion, itself may be a consequence of gastric maldigestion. In other word, abnormal gastric chylous results in abnormal hepatic chymous and abnormal hepatic chymous results in abnormal humors (Figure 2). It should also be noted that even a healthy liver does not have the ability to convert abnormal chylous into normal chymous and then normal humors.[58]

The most important point is that due to the close relationship between different organs that contribute to the digestive chain, the impairment in each of the stages of digestion will ultimately cause production of abnormal humor. This abnormal humor does not have the quality to become the desired end product. In later stages of digestion, the abnormal humor affects the function of organs consuming it and furthermore may gradually cause dysfunction of the next responsible organ in the digestion chain.[59]

### Clinical Support

Based on the above hypothesis, to find the responsible organ causing hypercholesterolemia and to provide scientific basis for deeply studying the relation between body humors and hypercholesterolemia, a pilot study was designed to assess 10 patients with high plasma cholesterol. The sign and symptoms of the dystemperament (Sui' a Mizaj) of the main digestive organs (Stomach and liver) were obtained. Symptoms which according to them the medical history was taken, were derived from the traditional diagnostic and treatment books, like The Qanon of Medicine of Ibn Sina (Avicenna),[60] the Kholasatol Hekmah,[61] the Moalejate Aghili,[62] the Exir-e-Azam of Hakim Azam Khan,[63] the Sharhe Asbaab-o-Alaamaat of Samarghandi,[64] the Zakhirah-E-Kharazm Shahi,[65] Al-Tasrif Le man ajeza an Talif[66] and the Asbab va Alayem.[67] Subsequently the data was tabulated and arranged so that full conclusions could be gained.

## Results

Our study indicated that only some of the patients had hepatic dystemperament (Sui' a Mizaj) and surprisingly all of the patients had gastric dystemperament. The significant difference between the results of gastric and hepatic dystemperament in our patients, suggests that the responsible organ involved in hypercholesterolemia is the stomach. Furthermore; the hepatic dysfunction, if not considered to be a consequence of gastric failure, will be the second responsible organ. Results of this study provide information for using Iranian traditional medicine in clinic and developing Iranian traditional medicine.

## Discussion

With reference to Iranian traditional medical texts, one can reach the basic point that organs involved in the process of digestion, particularly the stomach and the liver play the most important role in determining the blood composition. However, the proper function of stomach as the first step involved in the digestion chain should be emphasized. If the gastric digestion is impaired, the rest of the organs involved in digestion like the liver, blood vessels and tissues cannot be provided with the necessary qualified raw material (chylous) and therefore may suffer dysfunction, thus causing abnormal product formation such as abnormal humors and blood composition.

It can be concluded that the first and best action in the treatment of hypercholesterolemia is to treat the stomach dystemperament (Stomach Sui' a Mizaj) and if then the disorder persists, the dystemperament of the liver should be considered. Further clinical study is recommended to investigate this issue.
